# Bournemouth

**Published:** 1889-03

**Authors:** Alfred J. H. Crespi

**Affiliations:** Wimborne


### BOURNEMOUTH.

BY

ALFRED J. H. CRESPI,
*Wimborne.*

THE official opening of the Direct Bournemouth Railway is a good text for a description of that rising West Hants watering-place. Bournemouth has been growing in favour, and competing vigorously with other and older towns over which it has unquestionable advantages, the chief being its comparative newness. Some watering-places like Weymouth have had an existence of several generations, and their older streets are narrow, crooked, and unattractive; but that does not apply to Bournemouth, which has sprung up in the lifetime of people still living. The roads are straight, excellent, and broad; its side streets wide and mostly in good order; and the *tout ensemble* has a picturesqueness rarely found in English towns. Moreover the area covered is extensive, and the houses are well built, with ample space around them.

The bright climate is one of its chief advantages, and rightly so. Nowhere, however, in the United Kingdom is the winter warm, agreeable, and dry; nowhere can invalids sit out day after day. Nevertheless, there are wide differences, and the southern and south-western places have decidedly less cause for complaint than those in the north. For a British health-resort, Bournemouth is greatly favoured, and has many attractions for invalids who cannot go abroad to winter. With the rapid cheapening of continental travel, even our most fortunate health resorts are, however, feeling foreign competition keenly. I shall lay before the reader some valuable tables of temperature and other climatological observations, from which he will see the exact position of Bournemouth.

TABLE I.

EXTRACTED FROM MR. MARRIOTT'S TABLES.

| 1885. | ABSOLUTE. | | Mean Temperature. | Range. | ANNUAL. | | | Relative Humidity. |
|---|---|---|---|---|---|---|---|---|
| | Max. | Min. | | | Cloud. | Rainy Days. | Inches. | |
| Alston ... ... | 80.4 | 5.1 | 42.4 | 75.3 | 7.3 | 225 | 42.26 | 87 |
| Cheadle ... ... | 82.8 | 21.0 | 45.6 | 61.8 | 6.7 | 201 | 30.25 | 87 |
| Bournemouth ... | 82.7 | 21.3 | 48.8 | 61.4 | 5.9 | 164 | 27.26 | 81 |
| Torquay ... ... | 86.3 | 24.8 | 49.6 | 61.5 | 6.4 | 176 | 32.05 | 78 |
| Guernsey ... ... | 82.8 | 29.1 | 51.4 | 53.7 | 7.0 | 169 | 30.42 | 83 |
| 1886. | | | | | | | | |
| Alston ... ... | 77.7 | 2.0 | 42.5 | 75.7 | 6.9 | 229 | 63.2 | 83 |
| Cheadle ... ... | 78.8 | 18.2 | 45.9 | 70.6 | 7.1 | 213 | 36.41 | 87 |
| Bournemouth ... | 85.2 | 21.4 | 49.5 | 63.8 | 6.0 | 181 | 33.59 | 82 |
| Torquay ... ... | 82.3 | 24.3 | 49.6 | 58.0 | 5.9 | 193 | 37.75 | — |
| Guernsey ... ... | 78.1 | 29.2 | 51.5 | 58.9 | 6.7 | 198 | 40.25 | 86 |

TABLE II.

EXTRACTS FROM MR. GLAISHER'S TABLES FOR 1886.

| | | ABSOLUTE. | | Range | MEAN. | | | Cloud. | Rainy Days. |
|---|---|---|---|---|---|---|---|---|---|
| | | Max. | Min. | | Max. | Min. | Temperature. | | |
| Bournemouth... | 1st Quarter | 57.5 | 21.5 | 36.0 | 42.7 | 32.6 | 37.6 | 6.1 | 50 |
| Wolverhampton | ,, ,, | 60.1 | 13.4 | 46.7 | 39.9 | 28.8 | 34.3 | 8.0 | 48 |
| Bournemouth... | 2nd ,, | 75.2 | 30.0 | 45.2 | 58.7 | 44.6 | 51.0 | 5.3 | 39 |
| Wolverhampton | ,, ,, | 73.9 | 27.0 | 46.9 | 58.5 | 40.7 | 48.0 | 7.8 | 43 |
| Bournemouth... | 3rd ,, | 85.2 | 37.9 | 47.3 | 67.6 | 52.9 | 59.6 | 4.6 | 30 |
| Wolverhampton | ,, ,, | 81.8 | 34.4 | 47.4 | 66.3 | 49.0 | 56.5 | 6.6 | 35 |
| Bournemouth... | 4th ,, | 69.6 | 23.1 | 46.5 | 51.9 | 40.6 | 46.2 | 5.6 | 62 |
| Wolverhampton | ,, ,, | 70.6 | 19.4 | 51.2 | 48.0 | 36.7 | 42.1 | 7.5 | 53 |

TABLE III.

COMPILED BY MR. WILLIAM MARRIOTT.

HOURS OF SUNLIGHT.

| | 1885 | 1886 |
|---|---|---|
| London ... ... ... ... ... | 1018 | 1003 |
| Bournemouth ... ... | 1524 | 1538 |
| Buxton ... ... ... ... ... | 983 | 679 |
| Ventnor ... ... ... ... ... | 1704 | 1683 |
| Blackpool ... ... ... | 1326 | 1239 |

These tables are on the authority of Mr. Glaisher, F.R.S., and Mr. William Marriott, the able Assistant-Secretary of the Royal Meteorological Society, and cannot be disputed. They dispose by the iron logic of inexorable figures of any claims for a Sicilian beauty of climate. They show that Bournemouth enjoys a climate *three* degrees warmer than Wolverhampton, with about the same range of temperature and the same number of rainy days, but with not quite such a low absolute minimum. The one respect in which Bournemouth eclipses the Midland towns is, in the greatly larger amount of sunlight: in other words, there are twice as many hours of sunshine at Bournemouth as at Cheadle or Wolverhampton; consequently, on many days when it is not tempting to be out in the Midlands, at Bournemouth outdoor exercise is sufficiently pleasant. The biting east wind at Bournemouth—a disagreeable feature of the climate of the south coast from Brighton to Penzance—may fairly be set against the small amount of fog near the sea.

The climate of the United Kingdom is remarkable for its equability: the ocean-breezes modify it in all districts pretty equally; so that between the South of England and the most northerly Scotch islands the difference of mean temperature for the year is barely six degrees; and, contrary to the impression entertained, however, July in the south is 11° Fah. higher than July in Uist, while the difference in the January temperature is scarcely appreciable. From the beginning of November to the end of March, longitude, not latitude, is the most important factor.

Bournemouth is the name given to a large, somewhat ill-defined area, covered more or less thickly with houses. The northern part is Parkstone, and is in the parish of Poole; then comes a long, narrow district of houses—Branksome, Bournemouth West and Bournemouth East, and, still more to the east, Boscombe. Indeed, from Poole to Christchurch—a distance of nine miles—a good deal of scattered building exists. As Bournemouth is not incorporated, it is not easy to speak with certainty as to the population: 30,000 is the figure often given; but this probably includes parts of Poole and Christchurch.

The main portion of Bournemouth is curiously shaped; it is long and narrow, extending three miles along a straight, handsome, main thoroughfare, the old Christchurch road. The town, unlike many other seaside watering-places, is not exactly on, but at the distance of half-a-mile from the sea; at some parts approaching nearer, at others being farther. From no part of the town can the water be seen, although a few houses on the east and south cliffs look down on the beach, with its bustling crowds, and the magnificent new pier. No road extends along the sea, though one or two lead to it; no stately terrace or crescent looks over the sea; and in this matter the contrast between Bournemouth and some other watering-places is decided. To the stranger there is, consequently, the great charm of novelty. Bournemouth is singularly favoured in one respect—

indeed, I do not know any other health-resort to compare with it;—it is, the cheapness and inexhaustible variety of its sea-excursions. Without harbour, without shelter, it seems the very last town to have such unrivalled advantages; but then only four miles off is the capacious and convenient harbour of Poole, and, early and late, steamers ply from the pier at Bournemouth to Yarmouth, the Needles, Ryde, Ventnor, Cowes, Swanage, Torquay, and, though rarely, to the French coast. A guinea ticket entitles the holder to all the shorter excursions for the season: this is remarkably reasonable.

Another feature is the churches, far in excess of the requirements of the scanty population; and in the superabundance of places of worship it resembles Torquay and Brighton. Only one, St. Peter's, has great architectural pretensions: it is certainly one of the finest in the diocese, and is striking from its great size, lofty and graceful spire, and abundant handsome ornamentation.

Bournemouth is not easy to describe: with no principal railway-station, with scarcely any centre like most inland towns from which to start, and with no salient features, it does not lend itself to the requirements of the descriptive writer. It lacks splendid natural attractions: it has no Derwent Parade like Matlock; no splendid Promenade or Park like Cheltenham; no Jephson Gardens like Leamington; and no Pulteney Street and Royal Crescent like Bath. The Gardens, however, are no mean set-off, and on sunny days are crowded with visitors. Though small, and not conspicuous for fine timber and splendid flowers, they are completely sheltered from the wind, and are abundantly furnished with comfortable seats. It is a singularly handsome, clean, well-built, attractive place, with a light, sandy soil, quickly drying after rain; many handsome shops, and not a few fine villas, to which innumerable additions are being made.

Its pine-woods are its special feature. The soil being dry and sandy, is not well suited to deciduous timber; but pines flourish, and many side streets, especially on the East Cliff, are well shaded and extremely pretty, and in those places where the builders have not been energetically at work, glens and chines remain that present a strikingly pretty appearance, totally unlike anything else which I have seen. The rhododendrons, too, in June are very fine, and the display of blossoms magnificent. Branksome Chine is especially famous for its luxuriousness at that pleasant season.

The town abounds in hotels, lodging and boarding houses, and prices ought not to be high. Complaints are rife though as to the charges, and, I am sorry to add, many visitors have had cause to complain. At ordinary seasons hundreds of rooms are to let, and lodging-house keepers are vociferous in their complaints that the place is empty; and as no palatial terrace faces the sea, and no broad and magnificent promenade lays itself out for the opulent, no part of the town should demand exorbitant rents, and one can only regret the imprudence of lodging-house keepers.

Amusements are not abundant—at least, so residents say—and the theatre, one of the prettiest and most beautifully arranged I have ever seen, has unfortunately been converted into a room for miscellaneous entertainments. Of land excursions the favourites are, Wimborne, famous for its magnificent and unique Minster, with its central and west towers and curious chain library; and the New Forest. During the summer, carriages crowded with excursionists ply many times a day to Wimborne and to

Rufus' Stone, the latter twenty miles off. The most beautiful country, the picturesque Park and White Farm at Crichel, and the superb Museum at Rushmoor, can only be reached from Wimborne, between which and Bournemouth there is a service of fifteen trains up and as many down a day. Christchurch Priory is also very grand, and in size, massiveness, and singularity, has no nearer rival than Salisbury Cathedral, and Romsey Abbey; the last is not easy to reach, though its splendid proportions and unique architecture make an impression never effaced.

But Bournemouth is a health-resort: and for what diseases is it good, when is it in season, and who should go there? Enquiries are readily answered: it is always in season, and good in all complaints; in truth, many visitors are always there. Although reckoned a winter resort, it is universally a summer favourite. In July it is fairly full; but in August and September it is literally crowded, and were prices more reasonable few lodgings would be empty. In winter the place is practically deserted, though less so than in spring; at any rate, this is what residents say. It is not judicious to call a place the English Nice or Mentone, it makes strangers expect too much; and to promise immunity from cold and other climatal disagreeables is still less prudent. With March the weather often becomes bright, and brief spells of pleasant weather may be expected. These increase in length and frequency through April, and from the early part of May to the middle or end of August grounds of complaint are few. The autumn, after the break-up of the weather in October, is often dull and wet, with frequent mild, soft, and humid days, on which exercise is pleasant enough—foxy days sailors call them—the precursors of gales of rain and wind.

Bournemouth is charming, and rapidly growing in popularity and favour—rather heavily handicapped though by the infrequency of trains on the Somerset and Dorset: there are only three good trains to Bournemouth in the afternoon on that line, and not one in the morning; while from West Bournemouth only three good trains in the morning set off for the north, and not one in the afternoon. It is startling, but true, that one has to leave Bristol at 6.20 a.m. to reach Bournemouth at 11.15—five hours to traverse sixty-four miles; while one cannot leave Bournemouth in the afternoon for Bristol or Bath by any train travelling at good speed, five hours being again required. Much is expected of the Direct Bournemouth Railway from Brockenhurst to the East Station, which misses the angle at Ringwood and avoids the difficult and tedious route from that quaint old town to Bournemouth. Should the new loop be well worked, it will increase the advantages of the town, and attract many more visitors from the North and from Town. Other urgent improvements are, doubling the whole Somerset and Dorset system, and readier access to the North, with fewer delays at Bath and Templecombe; and more amusements of a high order. Visitors must not be regarded as mines of wealth; most, indeed, in these hard times, are only just able to pay their way with economy. The rich few are everywhere welcome; but Bournemouth must not forget the poor millions.

